# A global analysis of portion size recommendations in food-based dietary guidelines

**DOI:** 10.3389/fnut.2024.1476771

**Published:** 2024-11-19

**Authors:** Fanny Salesse, Alison L. Eldridge, Tsz Ning Mak, Eileen R. Gibney

**Affiliations:** ^1^UCD Institute of Food and Health, University College Dublin, Dublin, Ireland; ^2^Insight Centre for Data Analytics, University College Dublin, Dublin, Ireland; ^3^Nestlé Institute of Health Sciences, Nestlé Research, Lausanne, Switzerland; ^4^Nestlé Institute of Health Sciences Singapore Hub, Nestlé Research, Singapore, Singapore

**Keywords:** food-based dietary guidelines, portion size, dietary recommendations, dietary habits, food groups, healthy diet

## Abstract

**Objective:**

Since large food portion sizes (PS) lead to overconsumption, our objective was to review PS recommendations for commonly consumed food groups reported in Food-Based Dietary Guidelines (FBDGs) globally and to assess variation in PS across countries and regions.

**Methods:**

Consumer-oriented FBDGs from the Food and Agriculture Organization (FAO) online repository were used to evaluate dietary recommendations, PS and number of portions for common food groups. Guidelines were classified for each group as qualitative, quantitative, or missing. A standardized approach was applied to convert PS recommendations given as household measures, cup equivalents, pieces and other measures into grams for cross comparison. Variation of recommended PS of common food groups within and across regions was examined.

**Results:**

Among 96 FBDGs, variations were found both across and within regions. At a regional level, the highest median PS recommendations were seen in Europe for Meat, Fish and Pulses, in the Near East for Dairy products, and in Africa for most grain-based foods. Recommendations for Fruits and Vegetables showed the highest consistency across FBDGs worldwide, whereas guidance on Meat, fish & eggs and Cooked cereals/grains showed discrepancies in the classification of foods into categories, as well as in the number of portions per day.

**Discussion:**

While some variation in PS recommendations across countries can be expected due to cultural and regional dietary practices, inconsistent definitions to refer to a portion and varied derivation methods may further produce discrepancies. Harmonizing development methods for FBDG could help establish more consistent reference portion sizes and therefore provide clearer guidance to consumers.

## 1 Introduction

Public health bodies regard food-based dietary guidelines (FBDGs) to be a critical tool to promote healthy dietary habits and reduce the incidence of non-communicable diseases (NCDs). As such, FBDGs have been adopted by around 100 countries globally (1). FBDGs aim to translate the latest scientific evidence into practical food-based guidance for consumers, and therefore they should provide recommendations on both the types and amounts of foods and beverages that should be consumed to meet nutrient requirements, maintain a healthy weight, and prevent chronic diseases (2, 3). Their development typically involves multiple regional stakeholders and considers cultural, social, and economic factors that may affect food choices (4–6). Central to the guidance on the type and amounts of food and beverages to consume is the concept and use of portion sizes (PS). A “portion” is generally referred to as the amount of food that an individual is recommended to consume on one eating occasion (7, 8). PS can be described in grams, as food unit (e.g., one apple, one slice of bread), or with reference to common household measures, such as cup, spoon, plate or others (9). Alongside PS information within FBDGs, a recommended number of portions per day for each food group is often given.

Furthermore, consumers are routinely exposed to a diversity of messages concerning amounts of foods to consume, particularly in countries with labeled serving sizes (SS). While often used interchangeably with PS, SS are reference amounts for consumption, usually provided in grams or standard measures by manufacturers on packaged food products (10, 11). Within each food group, multiple servings can be consumed at one setting in a “portion”. PS can in fact be multiples of a single SS recommendation (e.g., one portion of pasta might contain 2–3 servings of the 8–10 recommended servings to be consumed a day). Although referring to different concepts, consumers often interpret labeled SS as a recommended serving for dietary guidelines rather than as typical consumption units (11). The lack of clarity between PS and SS can therefore result in a misinterpretation of dietary recommendations for consumers (11–14). In Europe for example, despite numerous age-appropriate dietary recommendations, a lack of consistent PS recommendations has been recently highlighted (9, 15).

The focus of this paper will be on PS, as used within FBDGs. PS are considered an important factor influencing food intakes and several studies have highlighted their impact on nutrient and health outcomes (16, 17). As PS for many foods have reputedly increased over the past decades, their increase, alongside other changes in food intake and lifestyle, has been linked to the global rise in obesity rates (12, 18–20). Indeed, overweight and obesity result from an imbalance between energy intake and energy expenditure (21, 22), and exposure to larger PS has been directly shown to lead to increased energy intake (known as the portion-size effect) (23, 24). A systematic review of 72 studies found that larger PS were associated with higher energy intake, increased body weight, and a higher risk of obesity (25). In 2014, Zlatevska et al. found that for certain foods, doubling the PS served led to a 35% increase in consumption (10). Adequate and consistent PS guidance could therefore play a crucial role in weight management (18, 19, 26), and the creation of harmonized standard portions for main food groups is considered relevant to improve information to consumers (27).

FBDGs, which include guidance on both the type and amount of foods (2), represent an opportunity to provide suitable PS guidance to populations. The introduction of regional (e.g., the Nordic Nutrition Recommendations (28)) and more recently global guidelines (e.g., the Planetary Health Diet (29)), underscore the ongoing efforts to establish consistent nutritional standards across geographies. However, these guidelines mostly focus on dietary patterns and total intakes per day, not on recommended portions of specific foods. While noted by several in the scientific community as being an important opportunity in providing cohesive nutritional recommendations, harmonization of PS recommendations is yet to be addressed (30).

Current literature shows variation in the PS recommendations provided by FBDGs within regions. A recent review of food PS in European FBDGs found heterogeneity in the attention given to PS recommendations, as well as a notable variation in the gram amount recommended for many staple food items (27). At a global level, little is known about recommended amounts of specific foods in FBDGs, as most studies to date have been limited to certain countries (4) or examined other aspects of the FBDGs, such as sustainability (31). The objective of the present study is to review PS recommendations for the most commonly consumed food groups in dietary guidelines globally and to assess variation across global regions. This work will form a basis for understanding commonalities and discrepancies in the ways that PS are derived and used by public health bodies in FBDGs worldwide.

## 2 Materials and methods

### 2.1 Documents screening

The Food and Agriculture Organization (FAO) online repository of FBDGs (1) was accessed between July 1st, 2023 and July 12th, 2024 to obtain a list of countries with published FBDGs. All countries listed on the FAO repository were considered for inclusion in the study. An additional web search was also systematically conducted to capture the latest/most updated versions of each country’s FBDGs, as well as additional background documents in the gray literature, using the following keywords: “[country] food-based dietary guidelines AND [scientific report OR scientific development].” All documents related to the listed FBDGs were accessed and downloaded for consideration, regardless of language. Google Translate was used to read documents written in any other language than English, French or Spanish.

The most recent version of all relevant documents was reviewed. For each country considered in this analysis, guidelines and recommendations aimed at the general healthy adult population were considered. The analyses were restricted to adult FBDGs only, therefore recommendations for infants, children, teenagers, elderly, pregnant and breastfeeding women were excluded. With respect to data extraction, a hierarchical process was employed. Consumer-targeted information was considered, rather than materials intended for healthcare professionals (HCPs) or scientific background documents, as this review aimed to evaluate the messages directly communicated to consumers. When multiple documents were available for consumers, the most comprehensive one was used for data extraction and cross-checked against any additional documents.

### 2.2 Data extraction and analysis

For each FBDG the following information was considered. First, we categorized the recommendations relating to food intake as either quantitative, qualitative, or absent. Quantitative recommendations consisted of a portion or serving size (e.g., “a medium fruit,” “120 grams” or “2 apricots, 2–4 pineapple slices, 1 good handful of small fruits”, …), number of servings per day (e.g., “eat dairy 3 times a day”) or a total amount to consume per day or per week (e.g., “eat a handful of nuts each day,” “eat 400 g of vegetables per day”). Qualitative recommendations referred to unquantifiable messages (e.g., “eat a variety of fruits,” “reduce red meat consumption”). If both qualitative and quantitative messages were given for a same food group, the recommendation was considered quantitative. If at least one food within a particular food group was mentioned, this food group was considered having a recommendation. For each food group, information on the portion size(s) for each food listed, and how many portions of this food were recommended to be consumed, either daily or weekly as appropriate, were collected. Specific details on this process for each food group are provided below.

Nine commonly consumed food groups were considered in our analysis, namely Fresh fruits, Vegetables, Grains, roots & tubers, Dairy, Meat, fish & eggs, Pulses, Nuts & seeds, Fats & oils, and Sugar & sweets. When a number of portions was given as minimum or maximum per day, that value was recorded (e.g., “at least 5 fruits and vegetables per day”). When given per week, recommendations on the number of servings were divided by 7 to obtain a daily value. When different recommendations were provided for specific population groups (e.g., for men and women separately), the detailed information was used, and the average was reported. In the case of a recommended value grouping more than one food category (e.g., fruits/vegetables, meat/fish/pulses), the number of servings was divided and split proportionally to the number of categories.

To determine the portion size of each of the food groups considered, a standardized approach was applied, where all PS recommendations (e.g., household measures, cup equivalents, pieces and other measures) were converted to grams. When information was provided as gram amounts at an overall food group level no conversion was necessary. If different examples of foods were given within a food group, the average recommended portion (g) of the individual food values was calculated for the food group. In the case of PS recommendations given in other units (e.g., cup, food item, and tablespoon) these were converted to a gram equivalent using two sources: the Food Portion Sizes version 3 book (32) and the USDA’s Food and Nutrient Database for Dietary Studies (FNDDS) 2017–2018 (33). When both sources provided a gram equivalent for the food, an average was computed. When only one had an equivalent, then its value was used. A visual aid tool (34) was used to convert recommendations provided in other common units (e.g., hand, palm, and plate). When none of these resources provided an equivalent and it was not possible to calculate a conversion, no value was included. However, it is important to note that this was uncommon and only affected 15 foods, which are detailed in Supplementary Table S3. If the document contained recommendations for several daily energy levels, the values corresponding to a medium activity level were considered. When a range of values was provided instead of a single amount, the mid-point of the range was reported.

In addition to the above, specific rules were applied for each food groups, as detailed below:

Fresh fruits: fruit juices, dry fruits, and coconut water were excluded from the PS calculations, to enhance consistency. For the PS recommendation “a medium fruit”, the average of the following medium fruits was applied: banana, apple, pear, peach, orange, mandarin. For the PS “1 cup of fruit” (or multiple thereof), the average of the value for 1 cup of the 10 most recommended fruits was applied (apple, banana, orange, watermelon, pineapple, grapes, mango, pear, papaya, plum).

Vegetables: 3 subcategories were considered: Vegetables (unspecified), Vegetables (excluding green/leafy) and Green/leafy vegetables. To account for variability in the way that vegetables can be eaten, if unspecified, the calculations considered an average of the raw and cooked weight of vegetables (when applicable). For the PS “1 cup of vegetables” or multiples thereof, the average of the value for 1 cup of the 10 most recommended vegetables from FBDGs was applied (tomato, carrot, lettuce, cucumber, cauliflower, pepper, cabbage, pumpkin, okra, and green/leafy vegetables). When two specific recommendations were provided for “Vegetables (excluding green/leafy)” and “Green/leafy vegetables”, the average of values for spinach, cabbage, broccoli and lettuce were considered in the green/leafy subcategory and excluded from the other one. While potatoes and other starchy roots were associated with vegetables in some FBDGs for the number of portions per day, they were excluded from the PS calculations for this food group and were considered as a subgroup of the Grains, roots & tubers food group instead.

Dairy: analysis of PS recommendations was performed for Milk & plant-based dairy alternatives, Yogurt & fermented dairy, and Cheese. 1 ml of milk was converted to 1 g of milk. To convert milligrams of yogurt into grams, the density 1.080 was used as a factor (32). Plant-based dairy alternatives were included in the same category as dairy milk, as they were most often considered within this group in the FBDGs. Kefir, and other local fermented dairy products were classified together with yogurts. Curd was classified in the cheese category. Other dairy-based products (e.g., custard) were excluded from the calculations.

Grains, roots & tubers: PS recommendations were split into the following subcategories: Bread, Cooked cereals/grains, Potatoes/starchy fruits & vegetables, and Ready-to-eat (RTE) breakfast cereals/muesli. Values for cereals were considered as cooked unless specified raw. Where a value for raw cereals was provided, this was converted to a cooked value using an average of the conversion factors for rice, pasta and noodles (2.0), as found in the USDA Food Buying Guide for Child Nutrition Programs (35).

Meat, fish & eggs: for the PS analysis, the following subcategories were considered: Meat, Fish & shellfish, and Eggs. When unspecified, 1 medium egg was considered to weigh 50 g. All meats (e.g., beef, pork, chicken, goat etc.) were grouped into a single category. Similarly, fish and shellfish which were also classified together (“Fish & shellfish” category).

Pulses: similarly to cereals, PS recommendations provided were considered cooked by default. No value to convert pulses was available in the USDA Food Buying Guide for Child Nutrition Programs (36), so we used the average of the values observed in the FBDGs which provided both raw and cooked values: 2.5, based on Afghanistan (2.5), Austria (2.1), Germany (1.8), Malta (2.0), Portugal (3.2), Spain (3.1), Turkey (2.6). When a recommendation of “1 glass” of pulses was given, it was converted in the same way as 1 cup. Soy products (e.g., tofu) were excluded from this category. Although they were initially reported in a separate category, only 9 FBDGs provided a PS for them, therefore the results are not included in this paper.

Nuts & seeds: this category included all types of tree nuts, ground nuts, and seeds. PS analysis excluded peanut butter and other similar pastes, olives, avocado, and lotus seeds. However, it is worth noting that these products were sometimes associated with Nuts & seeds on food pyramids and therefore are included in the recommended number of portions per day.

Fats & oils: includes oils, butter, and in some FBDGs other products as mentioned above in the nuts and seeds section.

Sugar & sweets: includes sugar, honey, jam/jelly, sweet snacks (candies, biscuits/cakes, etc …), chocolate.

The categories Fats & oils and Sugar & sweets were considered for the presence of quantitative/qualitative/no recommendation and number of portions per day, however, due to the low number of amount recommendations, they were excluded from the PS analysis.

All data were extracted manually, and stored on Microsoft Excel (Microsoft Office, V.2401). For each FBDG, portions were converted into gram amounts for each food, and food group. Quality checks were conducted by FS (the lead author) and PS values were reviewed by all team members. Outliers were identified and values were discussed within the research team. Three values were excluded from the calculation, as they were deemed implausible from a dietary intake perspective (e.g., in the Mexican FBDG, the recommendation for vegetables included a “1.5 raw cabbage” which when converted to a gram amount represented a PS of 1,050 g (700 g per cabbage x 1.5)). Data was extracted individually for each country, and summary for global regions was obtained by determining medians, standard error of mean (SEM) and minimum and maximum value for each food group by FAO global regions.

## 3 Results

### 3.1 Countries and regions

FBDGs from a total of *n* = 100 countries were listed on the FAO repository at the time of data collection (July 2023 to July 2024). Of the 100, three FBDGs were excluded from the analysis as some documents could not be accessed at the time of data extraction (Iran, Nepal, United Arab Emirates). One other country (Cambodia) was excluded, as only recommendations for children were available. Therefore *n* = 96 countries were included in the analysis: *n* = 2 in North America, *n* = 11 in Africa, *n* = 34 in Europe, *n* = 16 in Asia, *n* = 29 in Latin America and the Caribbean (LAC), and *n* = 4 in the Near East. The list of the included FBDG for each region, as well as the access link to their material, can be found in Supplementary Table S1.

### 3.2 Type of recommendations provided within FBDGs

Table 1 provides the frequency of quantitative or qualitative intake recommendations, or lack thereof, for food groups considered within this analysis. Globally, Fruits, Vegetables, and Meat, fish & eggs were the food groups for which quantitative recommendations were most commonly provided (Table 1). Fewer guidelines provided quantitative intake recommendations for the food groups Nuts & seeds, Fats & oils, and Sugar & sweets. North America and LAC were the regions with the lowest proportion of quantitative portion size guidance. As an example, within the LAC region for the food group Grains, roots & tubers, 48% of FBDGs provided quantitative messages, which corresponds to 15 countries not mentioning specific amounts (Table 1). With respect to Sugar & sweets, only a few countries provided quantitative recommendations, with 54 FBDGs providing some qualitative guidance, generally to limit consumption. Since the Canadian FBDG document provided qualitative guidance only, analyses of quantitative recommendations for this region are represented exclusively by the American FBDGs.

**Table 1:** Mean percentage of quantitative^1^ and qualitative recommendations for each food group provided in consumer FBDGs, by FAO region.

Region	*n* FBDGs	Fresh fruits			Vegetables			Grains, roots & tubers			Dairy			Meat, fish & eggs			Pulses			Nuts & seeds			Fats & oils			Sugar & sweets		
		QN	QL	NM	QN	QL	NM	QN	QL	NM	QN	QL	NM	QN	QL	NM	QN	QL	NM	QN	QL	NM	QN	QL	NM	QN	QL	NM
North America	2	50	50	0	50	50	0	50	50	0	50	50	0	50	50	0	50	50	0	50	50	0	50	50	0	50	50	0
Africa	11	73	27	0	64	36	0	82	18	0	73	18	9	73	27	0	73	27	0	64	18	18	64	27	9	45	36	18
Europe	34	94	6	0	94	6	0	85	15	0	85	15	0	91	9	0	68	29	3	68	24	9	50	47	3	21	62	15
Asia and the Pacific	16	94	6	0	94	6	0	88	13	0	88	6	6	94	6	0	88	6	6	63	13	25	63	25	13	44	38	19
LAC^2^	29	66	34	0	66	34	0	48	52	0	59	41	0	66	34	0	59	41	0	28	62	10	41	59	0	34	66	0
Near East	14	100	0	0	100	0	0	100	0	0	75	25	0	100	0	0	100	0	0	100	0	0	0	100	0	25	75	0
Total	96	82	18	0	81	19	0	74	26	0	75	23	2	81	19	0	70	28	2	55	32	13	49	47	4	32	56	10

^1A message on either the number of servings per day, total recommended per day, or portion size were considered as quantitative intake recommendations. 2Latin America and the Caribbean. n, number of included food-based dietary guidelines; QN, quantitative recommendation; QL, qualitative recommendation; NM, not mentioned.^

### 3.3 Number of portions per day

Supplementary Table S2 displays the recommended number of portions per day for each food group, at a global level (global median) and per global region. Fourteen consumer FBDGs did not include any recommendation for the number of portions to be consumed per day (Bahamas, Brazil, Canada, Dominica, Ecuador, Grenada, Guyana, Kenya, Namibia, Nigeria, Saint Lucia, Sierra Leone, Thailand, and Viet Nam) and 7 provided portion recommendations per day for only one food group (China, North Macedonia, Panama, Peru, South Africa, Sweden, Uruguay). In many instances, food categories were grouped into a unique recommendation. The most observed combinations were Fresh fruits/Vegetables (23 FBDGs, of which 1 combined Fresh fruits/Vegetables/Legumes) and Meat, fish & eggs/any other group (23 FBDGs, of which Meat, fish & eggs/Dairy: 6 FBDGs; Meat, fish & eggs/Pulses/Nuts & seeds: 7 FBDGs; Meat, fish & eggs/Pulses: 7 FBDGs, and Meat, fish & eggs/Dairy/Pulses/Nuts & seeds: 1 FBDG). Other observed combinations included Pulses/Nuts & seeds (5 FBDGs) and Grains, roots & tubers/Pulses (4 FBDGs). As seen in Table 2, the range of the number of daily recommended portions is wider for some food groups than for others. The lowest variation was observed for Fresh fruits and for Vegetables, with median recommended numbers of portions per day spanning from 2 to 3 for Fresh fruits, with a coefficient of variation (CV) of 0.2, and range from 2.3 to 4.1 for Vegetables, with a CV of 0.3. The number of recommended portions for Meat, fish & eggs was low in countries which only specified an amount for Fish (Supplementary Table S2). The highest CVs were found for Pulses and Sugar & sweets, Pulses and Nuts & seeds (0.8). The highest recommended number of daily portions was found for Grains, roots & tubers, with a global median of 6 portions. African FBDGs had the lowest recommended number of daily portions per day for most food groups and showed a median recommendation below the global median for all of them.

**Table 2:** Median number of recommended portions per day for major food groups, by FAO region.

Region	Fresh fruits	Vegetables	Grains, roots & tubers	Dairy	Meat, fish & eggs	Pulses	Nuts & seeds	Fats & oils	Sugar & sweets
North America	2.00	2.28	6.00	3.00	4.86	0.21	0.71	-	-
Africa	2.25	2.75	4.75	1.50	0.85	0.90	1.00	1.00	0.75
Europe	2.50	3.00	7.00	2.50	1.29	0.50	1.00	2.00	1.00
Asia and the Pacific	2.00	3.25	6.00	2.00	2.50	0.95	0.60	5.00	3.75
LAC^1^	2.50	3.00	7.00	3.00	2.01	1.50	1.50	3.75	3.50
Near East	3.00	4.00	7.00	2.00	1.4	1.30	1.30	-	-
Global median	2.50	3.00	6.50	2.50	1.43	1.00	1.00	2.00	2.00
CV^2^	0.24	0.29	0.47	0.37	0.71	0.78	0.76	0.64	0.77

^1Latin America and the Caribbean. 2CV = Coefficient of variation.^

### 3.4 Portion size recommendations for the main food groups, considered by FAO global region

#### 3.4.1 Fresh fruits and Vegetables

Recommended portion sizes (PS) for Fresh fruits and Vegetables, grouped by FAO region, are presented as global averages in Table 3. Supplementary Table S3 presents these results detailed for each specific country considered within this analysis. Across global regions, the median recommendation for Fresh fruits remained relatively consistent, all ranging between 120 g in Europe and 139 g in the Near East, except for North America (154 g). Important variation could be observed within regions, with maximum values being 2 to 3 times higher than the minimums in Europe and LAC. The lowest values were observed in Indonesia (50 g), Republic of Moldova (68 g), Barbados (77 g). Regarding the Vegetables categories, *n* = 39 countries provided an unspecified PS recommendation. Other countries provided different recommendations for Green/leafy vegetables (*n* = 26) and Vegetables excluding green/leafy (*n* = 27). For example, all FBDGs in the Near East region recommended specific PS for Green/leafy and non-green/leafy vegetables. When PS for the unspecified vegetables were calculated, they ranged from 50 g in the Netherlands to 204 g in Argentina. Values were found to be most consistent in Asia and the Pacific, with a minimum value of 75 g (Australia) close to the maximum of 102 g (Afghanistan). Despite some regional variations, the medians were consistent across regions and spanned from 80 g in Africa to 100 g in Europe, Asia and the Pacific, and LAC. A larger variation was observed in the recommended PS for the vegetable category which included Green/leafy vegetables only. Regarding the category excluding green/leafy vegetables, Asia and the Pacific and Africa showed the lowest regional medians (82 and 87 g, respectively) and Europe and the Middle East the highest (119 g). Values were particularly spread around the median in Africa (SEM = 20.2 g) and a 115 g gap existed between the highest and the lowest recommended PS (respectively 140 g in Sierra Leone and 25 g in Ethiopia). PS values for Green/leafy vegetables were in all regions lower than those for non-green/leafy vegetables and for unspecified vegetables. The global median PS for this category was 70 g. In each region, the set of recommended PS was noticeably spread around the median in each region, as shown in Table 3 by high SEM values, particularly in Asia and the Pacific and in Africa.

**Table 3:** Portion size recommendations in grams for Fresh fruits and Vegetables in FBDGs, by FAO region.

		North America	Africa	Europe	Asia and the Pacific	LAC^1^	Middle East	All
Fresh fruits (g)^3^	*n* FBDGs	1	7	28	9	17	4	66
	Median	153.5	130.6	119.5	124.0	134.5	138.7	127.6
	SEM^2^	–	7.5	5.2	10.8	4.8	11.9	3.1
	min	–	100.0	66.7	50.0	76.8	106.5	50.0
	max	–	162.5	162.0	150.0	150.0	157.6	162.5
Vegetables – unspecified (g)^4^	n FBDGs	0	3	21	5	10	0	39
	median	–	80.0	100.0	100.0	99.6	–	100.0
	SEM	–	27.5	8.8	5.1	11.8	–	5.7
	min	–	71.0	50.0	75.0	70.0	–	50.0
	max	–	157.7	200.0	102.2	204.0	–	204.0
Vegetables –excluding green/leafy (g)	n FBDGs	1	5	6	5	6	4	27
	median	128.3	86.7	118.8	81.6	100.4	119.4	100.4
	SEM	–	20.2	19.7	16.9	10.4	17.2	7.4
	min	–	25.0	75.6	46.3	91.3	81.6	25.0
	max	–	140.0	200.0	150.0	158.3	158.3	200.0
Vegetables – green/ leafy (g)	n FBDGs	1	5	7	5	4	4	26
	median	54.0	50.0	80.0	47.3	86.8	73.8	70.0
	SEM	-	24.6	16.4	16.7	31.3	10.4	8.6
	min	-	35.0	36.3	29.0	58.5	54.0	29.0
	max	-	164.4	164.0	125.0	199.1	94.5	199.1

^1^Latin America and the Caribbean. ^2^Standard error of the mean. ^3^Fresh fruits: excludes juices, coconut water, dry fruits (prunes, raisins, …). ^4^Vegetables: unspecified: includes all types of vegetables; excl. Green leafy: when a different recommendation was given for general vegetables and green leafy vegetables – this category excludes green leafy vegetables; green leafy vegetables: includes any green leafy vegetable as provided, or an average of the values for spinach, cabbage, lettuce and broccoli, if the types of green leafy vegetables were not specified.

#### 3.4.2 Grains, roots & tubers

The recommended PS for the Grains, roots & tubers food group are presented in Table 4, which shows a global median for this category of 90 g. Disparities in median recommendations were observed for Cooked cereals/grains, with variations in minimum and maximum amounts spanning from 30 g to 247 g, respectively in the Netherlands and in the Philippines. The lower values were often presented alongside a high number of recommended portions per day, ranging from 9 to 15 (Table 2). This may imply a recommended consumption of more than one serving per meal, however this is not specified. Within regions, recommendations were less consistent in Africa and in Asia and the Pacific, as per the high SEM values. African countries lacked specific recommendations for RTE breakfast cereals/muesli yet provided the highest PS recommendations for Bread and Cooked cereals/grains (142 g and 79 g, respectively). In contrast, the Near East FBDGs showed notably lower bread recommendations compared to other regions (median of 27 g versus 42 g globally), albeit with a recommended number of servings exceeding six per day, resulting in effectively doubling or tripling the actual portion size per eating occasion. The United States recommended a portion of 28 g. No country in Africa provided a recommendation for (RTE) breakfast cereals/muesli. The highest value (60 g) was observed in Germany. The lowest value was provided in the LAC region, with a PS of 18 g recommended in Costa Rica, and the lowest median (22.5 g) was observed in this region as well. With two recommendations of 36 and 43 g (in Oman and Lebanon, respectively), the Near East showed the largest median for RTE breakfast cereal recommendations. Finally, Table 4 displays the recommended PS for Potatoes/starchy fruits & vegetables. For this category, regional median values ranged from 100 g in Asia and the Pacific, to 140 g in Africa, with despite notable differences in minimum recommendations (50 g in Asia and the Pacific and 117.5 g in Africa). The median PS recommendation in Europe was almost as high as the African value (138 g), and the maximum amount recommended was also observed in this region (250 g in Germany).

**Table 4:** Portion size recommendations in grams for Grains, roots & tubers in FBDGs, by FAO region.

		North America	Africa	Europe	Asia and the Pacific	LAC^1^	Middle East	All
Cooked cereals/grains (rice, pasta, noodles…)^3^ (g)	*n* FBDGs	1	4	21	9	10	3	48	median	74.5	142.3	85.0	97.5	90.0	78.2	88.0	SEM^2^	–	25.3	11.3	21.8	7.8	2.7	7.0
	min	–	79.0	35.0	30.0	74.5	77.5	30.0
	max	–	202.5	240.0	246.5	150.0	86.0	246.5
	Bread (g)	n FBDGs	1	6	20	7	11	3	48	median	28.4	78.8	47.8	50.0	39.6	26.9	41.1	SEM	–	24.2	4.7	21.1	3.1	3.4	5.2
		min	–	37.0	20.6	29.2	36.5	26.5	20.6
max		–	173.3	100.0	158.8	67.0	36.9	173.3
Ready-to-eat breakfast cereals/ muesli (g)		n FBDGs	1	0	11	3	2	2	19	median	28.4	–	30.0	30.0	22.5	39.5	30.0	SEM	–	–	4.3	4.7	4.5	3.5	2.7
		min	–	–	18.0	21.3	18.0	36.0	18.0
	max	–	–	60.0	37.5	27.0	43.0	60.0
	Potatoes/ starchy fruits & vegetables (g)	n FBDGs	0	3	17	7	12	0	39	median	–	140.0	138.0	100.0	115.0	–	137.5	SEM	–	11.0	13.8	17.0	12.3	–	8.4
		min	–	117.5	80.0	50.0	60.8	–	50.0
max		–	155.5	250.0	180.0	200.0	–	250.0

^1^Latin America and the Caribbean. ^2^Standard error of the mean. ^3^Values for cereals were considered as cooked unless specified raw. Where a value for raw cereals was provided, this was converted to a cooked value using an average of the conversion factors for rice, pasta and noodles (2.0), as found in the USDA food buying guide for child nutrition programs.

#### 3.4.3 Dairy products

As seen in Table 5, the PS recommendations for Milk/plant-based dairy alternatives were consistent throughout the world, with 3 regions showing a similar median recommendation of 222–222.5 g (Africa, Europe, LAC). The more notable variation was observed in Asia, where both the minimum and maximum values were found (100 g in India and 313 g in Malaysia, respectively). Overall, most values ranged between 200 and 250 g for the Milk PS, with 44 out of 53 FBDGs providing a recommendation within this range. However, recommended PS for Cheese showed considerable variation across countries, particularly in Europe and in Asia and the Pacific, where the values ranged from 17 to 152 g and from 15 to 100 g, respectively. The lowest values (<20 g) were found in Sri Lanka (15 g), Jamaica (16 g), and Belize, Costa Rica, and Iceland (17 g). On the contrary, values ≥100 g were observed in Albania, Austria, Republic of Moldova, and India. As seen in Table 5, the Near East and Europe showed the highest regional medians, while lower median PS recommendations were found in Southern regions (LAC and Africa). The global median was 39 g. Medians were highest for all dairy categories in the Near East and North American FBDGs, in comparison to other regions, with a higher variation for Yogurts and fermented dairy (245 g versus a global median of 182 g). Just like for Milk, the lowest median for this category was observed in Asia and the Pacific with a value of 124 g. The recommendations varied widely among African FBDGs with a SEM value of 53 g and a maximum PS recommendation observed in Ethiopia being almost 3 times larger than the minimum observed in Benin and Gabon (respectively 350 g and 125 g).

**Table 5:** Portion size recommendations in grams for Dairy in FBDGs, by FAO region.

		North America	Africa	Europe	Asia and the Pacific	LAC^1^	Middle East	All
Milk and Plant-based milk alternates (g)	n FBDGs	1	4	25	10	11	3	54	median	244.0	222.5	222.0	200.0	222.0	244.0	222.0
	SEM^2^	–	13.8	7.0	21.2	10.4	1.3	5.7
	min	–	200.0	125.0	100.0	122.0	240.0	100.0
	max	–	250.0	250.0	312.5	244.0	244.0	312.5
	Yogurt & fermenteddairy (g)	n FBDGs	1	4	21	6	11	3	46	median	245.0	162.5	170.0	124.0	188.5	245.0	181.8	SEM	–	53.0	9.1	19.3	17.3	4.3	8.2
min		–	125.0	125.0	100.0	56.7	232.0	56.7
max		–	350.0	259.2	202.5	245.0	245.0	350.0
Cheese (g)		n FBDGs	1	4	25	6	13	3	52	median	49.6	27.5	50.0	40.0	30.0	52.5	39.0	SEM	–	6.6	6.9	13.8	5.6	4.3	4.1
		min	–	20.0	16.7	15.0	15.6	45.0	15.0
	max	–	50.0	151.7	100.0	75.0	60.0	151.7

^1Latin America and the Caribbean. 2Standard error of the mean.^

#### 3.4.4 Meat, fish & eggs and Pulses

Table 6 groups PS recommendations for major Meat, Fish & shellfish, Eggs, and Pulses found in FBDGs. Europe had notably higher recommendations for Meat, Fish & shellfish, and Pulses, encompassing all major protein sources except Eggs, which generally provided recommendations of 1 egg per portion. Indeed, only 10 out of 43 countries recommended more than 1 egg per portion (with values ranging from 80 to 125 g). The range of recommended PS values was particularly wide in Europe for Meat (from 27.5 g in Portugal to 135 g in Greece) and Fish & shellfish (from 27.5 in Portugal to 200 g in Romania). The highest PS recommendation for Meat was that of the Greek FBDGs, at 135 g. In regard to Fish & shellfish, maximum amounts were given in the Republic of Moldova and Romania (200 g). In regard to Fish & shellfish, maximum amounts were given in the Republic of Moldova and Romania (200 g). Conversely, Near East FBDGs suggested low recommendations for Meat intake, with PS for Meat of 30 g in Lebanon and Oman. The Asia and the Pacific region emphasized high PS values for Pulses with a regional maximum recommendation of 240 g in Malaysia, despite a few countries providing a low recommendation (30 g in Bangladesh and India). The lowest regional median for this group was observed in North America and in the Latin American FBDGs (46 and 80 g, respectively) as well as the recommendations for Fish & shellfish (28 and 38 g, respectively). As mentioned in section 3.3, there were inconsistencies in how food groups were categorized within this group, with Meat, fish & eggs sometimes being treated individually, while other times being grouped under broader categories such as “animal foods”. Also, the units and frequency differed for many protein-rich foods. These inconsistencies made it challenging to discern a consistent approach to grouping foods across recommendations.

**Table 6:** Portion size recommendations in grams for Meat, fish & eggs and Pulses in FBDGs, by FAO region.

		North America	Africa	Europe	Asia and the Pacific	LAC^1^	Middle East	All
Meat^3^ (g)	n FBDGs	1	6	20	9	14	3	53	median	28.4	77.7	92.5	72.5	66.7	30.0	75.0	SEM^2^	–	2.1	6.5	7.0	5.5	15.0	3.6
	min	–	69.8	27.5	30.0	30.0	30.0	27.5
	max	–	85.0	135.0	82.0	98.0	75.0	135.0
	Fish & shellfish^3^ (g)	n FBDGs	1	6	23	9	9	3	51
	median	28.4	98.1	120.0	70.6	38.1	75.0	90.0	SEM	–	18.7	9.9	11.4	8.1	18.0	6.8
min	–	58.3	27.5	30.0	30.0	30.0	27.5
max	–	190.0	200.0	132.4	90.0	90.0	200.0
Eggs^4^ (g)	n FBDGs	1	6	18	8	12	2	47
median	50.0	65.0	52.5	50.0	50.0	50.0	50.0	SEM	–	9.4	6.5	10.7	0.3	0.0	3.4
min	–	50.0	50.0	50.0	47.0	50.0	47.0
max	–	100.0	125.0	120.0	50.0	50.0	125.0
Pulses^5^ (g)	n FBDGs	1	7	20	9	13	3	53
	median	45.8	95.0	127.5	100.0	80.3	90.7	92.5
	SEM	–	7.9	13.9	24.5	8.6	15.1	7.6
	min	–	75.0	46.0	30.0	46.0	46.0	30.0
	max	–	132.0	250.0	240.0	125.0	91.9	250.0

^1^Latin America and the Caribbean. ^2^Standard error of the mean. ^3^Values can be either cooked or raw, depending on the FBDGs. Most of the times no precision was provided as to raw or cooked. ^4^Eggs: 1 medium egg was considered to weigh 50 g. ^5^Pulses: values were considered cooked, except for when “dry” seemed to refer to raw rather than to opposite of fresh pulses (Switzerland), or when the value provided was deemed irrational to be cooked as very low (Estonia). In these cases they were converted to cooked using factor 2.5. Excludes soy products such as tofu, tempeh, etc.

#### 3.4.5 Nuts & seeds

The PS recommendations for Nuts & seeds varied across regions, with median values spanning from 13 to 30 grams (in LAC and Asia and the Pacific, respectively), as seen in Table 7. LAC, North America and the Near East generally had lower recommendations compared to Asia, where values tended to be notably higher. The two observed recommendations in the Middle East were 15 g (Lebanon, Oman). In LAC, most recommended PS were close to the median (within a 5 g above or below) with the exception of Argentina which provided a PS recommendation of 27 g. Notably high PS recommendations could be observed in Benin (50 g) and in Greece (40 g), with Africa and Europe showing the widest range of values, spanning, respectively, from 5 to 50 g and 11 to 40 g. Values were particularly inconsistent in Africa, as shown by a SEM of 6.4 g.

**Table 7:** Portion size recommendations in grams for Nuts & seeds in FBDGs, by FAO region.

		North America	Africa	Europe	Asia and the Pacific	LAC^1^	Middle East	All
Nuts & seeds^2^ (g)	*n* FBDGs	1	6	13	6	7	2	35	median	14.2	20.8	25.0	30.0	13.3	15.0	23.5	SEM^3^	–	6.4	2.4	3.6	2.3	0.0	1.8	min	–	5.0	11.3	15.0	8.0	15.0	5.0	max	–	50.0	40.0	35.7	27.0	15.0	50.0

^1^Latin America and the Caribbean. ^2^Excludes peanut butter, olives, avocado, lotus seeds (which were sometimes included in this food group in FBDGs). ^3^Standard error of the mean.

## 4 Discussion

This research aimed to examine portion size recommendations across food-based dietary guidelines (FBDGs) globally looking specifically at the provision of and variability within portion size recommendations. A total of 96 FBDGs were considered therefore to our knowledge, this paper represents the first global comparative analysis of food group portion size recommendations. This work highlights both variations and consistencies in recommended portion sizes, within and across food groups at a regional and global level.

Not all guidelines provided quantitative intake recommendations, with differences being particularly evident within specific food groups and across global regions. Apart from the North American region which was represented by only 2 countries, LAC provided the least quantitative recommendations within their FBDGs, whereas Europe provided the most quantitative recommendations. In all regions, Grains, roots & tubers was the food group with the highest number of recommended daily portions to be consumed, which is in line with other studies which highlighted grains as the food group representing the highest relative amount of food to be eaten (37). With respect to food groups with recommended PS, Fresh fruits & Vegetables were among the food groups with PS most often recommended. These findings are in accordance with previous studies, where messages encouraging consumption of fruits and vegetables were reported to be the most frequent in FBDGs worldwide (38). PS recommendations within these food groups were also the most consistent across the guidelines considered (3). On the contrary, the food group Sugar & sweets had the fewest quantitative recommendations and were more often mentioned alongside qualitative guidance, generally encouraging to limit or reduce their consumption. This is in line with existing recommendations from the WHO whereby a restrictive recommendation of less than 10% of dietary energy from sugar intake is given (39). Therefore, stakeholders developing FBDGs may have used the approach of recommending only limited amounts and infrequent consumption to be more relevant from a public health perspective than providing an actual amount for such a food group. Moreover, strategies to address sugar intakes have more recently focused on other approaches than FBDG recommendations, such as provision of personalized nutrition advice (40) or reformulation strategies (41–44). Furthermore, our work found that some FBDGs provided total amounts per day or number of portions per day, but not specific recommendations on a PS for some food groups. For example, the Vietnamese FBDGs provided monthly amounts to consume but gave no information on recommended daily food intakes.

The work presented here also identified disparities in the way food groups are defined, as well as the way that foods are classified into food groups. While commonalities existed (e.g., the frequent combination of Fruits and Vegetables, as a single food group, observed in many regions), discrepancies were equally notable, particularly regarding protein sources and animal foods. For example, in Latin American FBDGs, dairy products were often considered “animal foods” and recommended together with other sources of protein, such as meat, fish (e.g., in Venezuela, Saint Vincent and the Grenadines, or Grenada) whereas in other global regions, notably Europe and North America, dairy products were more commonly kept apart from other animal-based foods. The lack of consistency in defining food groups is a well-known issue in nutrition research, and initiatives have been taken to address coding of food data to bring uniformity, and comparability of datasets including use of FoodEx2 coding in EFSA Food Consumption Database (45) and other similar strategies (46, 47). This was also the focus of the work conducted within the FNS-Cloud project, which developed innovations and support for to address food and nutrition data federation across Europe (48). However, such work is mostly dedicated to data collection in the context of nutritional surveys (49) rather than for the development of nutritional recommendations. The learnings or the approaches taken in such projects may not have been considered within the context of FBDG to date.

While one can consider variation in a negative manner, it is important to understand the reason for variation, and embrace the fact that variation is both natural and needed. One plausible explanation for the variations observed could be the influence of cultural eating habits across different global regions (38, 50) as well the availability and access to specific foods, which will result in both different food groups and/or foods within these groups. This aligns with findings from Carruba et al. who examined recommended PS in European FBDGs and found an influence of cultural attitudes toward foods, which manifested in country/regional differences (27). Local knowledge of dietary and culinary habits is known to be essential for the development and use of FBDGs (2) and is therefore naturally reflected in the variation observed. For example, the large PS recommendations observed in Africa for subgroups Cooked cereals/grains, Bread and Potatoes/starchy fruits & vegetables can be explained by the general high share of carbohydrates in the regional diet, which represents about 70% of the daily energy supply (51). On the other hand, the high consumption of meat in Europe (52) seems to be leading public health bodies to recommend limiting its intake (53) and increase that of fish instead, as shown by the larger recommended PS of fish in many European FBDGs. To reduce the variability in our analysis, some specific foods were excluded from the calculations (e.g., coconut water excluded from Fresh fruits, or corn-based products excluded from Cooked cereals/grains). It is however important to note that in certain countries, these foods may be an important part of the diet. While the aim of this paper was to evaluate the commonalities and differences in FBDGs and provide an overview of the observed variations, currently ongoing research is further analyzing these variations to determine whether regional differences are significant. This will enable allow a deeper understanding of how local habits shape dietary guidelines.

When considering food groups and foods within food groups, the variation within the Meat, fish & eggs group is worth noting. Meat, fish & eggs were commonly combined into a “Protein group”, occasionally along with other foods with a high protein content such as Pulses or Nuts & seeds. With the emergence of sustainable dietary concerns (29, 54), as well as scientific evidence associating red and processed meat consumption with NCDs (55, 56), public health bodies are encouraging consumption of more plant-based foods. The importance of providing specific recommendations for meat and non-meat protein sources has been addressed in several recently developed European FBDGs (e.g., Denmark, 2021; France, 2019; Spain, 2022). However, it is difficult to understand if such messages are driven by nutritional or sustainability concerns, or both (3). In this analysis, the countries who introduced sustainability concerns did not necessarily recommend low PS for meat. In Europe for example, the Italian and the Dutch FBDGs extensively mentioned sustainability, however both provided recommended PS for Meat of 100 g, which is greater than the global median. However, the advised frequency of consumption in these documents was low. This highlights the importance of considering both the recommended PS and the number of portions per day or per week when assessing the sustainability of a diet. A review of plant-based diets and substitutions for animal-based foods in FBDGs recently highlighted an overall lack of recommendations for alternatives to meat and animal milk (31). The need for a reform of FBDGs, through the sustainability lens, has been stressed by Springmann et al., who pointed out the need for more specific recommendations including suggested minimum values for plant-based foods such as whole grains, nuts, and legumes, and stricter limits for red and processed meat and dairy (57). While no particular trend was identified between the year of publication of the guidelines and the combination of different sources of protein as food groups in our study, the recent issue of regional and global guideline documents which are mainly based on environmental aspects demonstrates the efforts to promote a shift of dietary habits toward sustainable consumption (28, 29).

The consideration of different baseline daily energy levels for the diet needs to be considered when comparing or considering variation across recommended portion sizes. While most FBDGs specified recommended intakes relevant to a 2000–2,200 kcal diet, variation was noted both within and across global regions. For example, the baseline energy level in the Zambian FBDG was 2,100 kcal, and that of the Ethiopian FBDGs was 2,700 kcal, representing a 25% difference across these 2 FBDGs within the same region. On the other hand, the energy level used in the Malaysian guidelines was 1800 kcal. Such variation does however not seem to affect the PS recommendation for each food group equally: in the case of Malaysia, the recommended PS are comparable for Fresh fruits and Meat, but vary widely for other foods such as Fish or Pulses. While some FBDGs explain how their recommended amounts help individuals meet energy and nutrient requirements (e.g., Malaysia, United States), others do not elaborate on whether their recommended values ensure that all calorie and nutrient needs are met (Hungary, Qatar). Future work could therefore focus on the standardization of the recommended PS to compare computed values based on a similar calorie requirement.

Variation in the recommended PS can also be linked to the method of derivation of the FBDGs. As mentioned above, guidelines exist to guide stakeholders in the development and implementation of the FBDGs and recommend following a stepwise approach to identify critical nutrients for the country population and select foods that are sources of these nutrients (2). This can be achieved in different ways, according to the resources available in each country. While some FBDGs are uniquely based on scientific consensus and daily energy requirements of the population (e.g., Philippines, Paraguay, Georgia, Kenya), others were derived through analysis of national consumption data and/or a diet modeling approach (Germany, Sri Lanka, Oman, United States). For European countries, EFSA has specifically emphasized the importance of modeling the effectiveness of FBDGs, which involves the use of nutrient intake data (58). In a 2018 review, Blake and colleagues highlighted that inconsistencies and deficiencies existed in the methods to review the evidence when developing FBDGs (59). However, this study only included 32 countries, and further research is still needed to fully understand the extent of these methodological gaps among FBDGs worldwide.

Addressing the terminology used with regards to PS is important. The terms “portion” or “serving”, which are often used interchangeably, even among the scientific community and HCPs, can be confusing for consumers, who are often exposed to SS on packaged foods, and then PS within FBDGs. In countries without regulations, SS are manufacturer’s suggestions and do not necessarily reflect recommendations from national guidelines. Further confusing the issue is that there is no distinction between the words “servings” and “portions” in many languages, so the labels would in fact reflect a portion (e.g., porzione (Italian), porcija (Lithuanian), porciones (Spanish), etc.). In FBDGs, lower PS values for Cooked cereals/grains or for Meat, fish & eggs were typically associated with higher recommended numbers of servings per day (for example in the Netherlands or in India). In these cases, the term “portion” was considered as including more than one serving, meaning the PS recommendation per meal was higher (e.g., the Indian FBDG recommend a 30 g PS for cooked grains, and 9 to 12 servings per day, meaning that 2 to 3 servings may be consumed within one meal, and the PS could therefore reach 90 g). This lack of consistency in the terminology used to provide PS recommendations is of concern and should be addressed to ensure an efficient delivery of the guidance to populations (13).

Discrepancies also exist between the guidelines provided for consumers and those produced for HCPs, and other experts. While emphasis was put on consumer-facing material, the different ways in which messages were included in FBDGs could lead to different interpretations of the recommended PS. Additionally, the variation which was detected between “theoretical” guidelines stemming from general scientific consensus and the actual translated recommendations provided to consumers often reflects a discordance on the approaches taken to disseminate nutrition messages (60). A characteristic example of this is the recommendation for Fresh fruits and Vegetables. Populations, particularly in Europe, are often advised to eat 5 fruits and vegetables a day (61). This originated from an arbitrary split of the WHO recommendation to consume at least 400 g per day, which were found to be beneficial for human health, into 5 portions of 80 grams (62). In practice, our calculations demonstrated the median PS recommendation for Fresh fruits was found much higher, for all studied regions, with the global median being of 126 g. Stakeholders in charge of developing FBDGs should ensure all messaging formats provide a consistent message to the consumer. Moreover, the inherent vagueness of certain messages may result in subjectivity of the interpretation (e.g., “a small fish”, “a medium plate”) and inevitably relies on social and cultural interpretations. Even when providing more detail, recommendations may be understood in different ways: fruits with or without the pit, rice or pasta raw or cooked, nuts with or without the shell. These limitations in the context of a scientific review highlight the potential difficulty for populations to efficiently translate the messages they are exposed to into adequate consumption patterns. The creation of consistent reference PS in line with dietary needs has already been indicated previously (27).

The FAO/WHO guidelines stipulate that behavioral and social sciences should be taken into account to enhance effectiveness of messages (2). The lack of harmonization in the way that PS recommendations are generally provided demonstrates that issues remain in considering the most practical way to provide dietary recommendations. Attention should be given to providing gram amounts for all foods as a reference, alongside the display of equivalent common household measures as visual / graphic messages are better understood by consumers. The WHO also notes that “consumers think in terms of foods rather than of nutrients” (2). The inclusion of reference PS within FBDGs therefore needs to be considered urgently as they provide practical guidance for consumers on the foods they should eat (27). Messages need to be communicated efficiently for populations to heed the FBDGs. Indeed, studies have shown that adherence to FBDGs is relatively low, with almost 40% of populations not complying with the recommendations, both in low and in high-income countries (63). Efforts are therefore needed to harmonize and strengthen FBDG messages in order to increase population awareness and use of FBDG PS recommendations (50, 64, 65).

This paper offers a global analysis of FBDGs, examining several food groups using a standardized approach to compare recommendations across major food categories and subcategories. However, some limitations are worth noting: the translations of some documents may have introduced interpretative bias, and the selection of a single document from multiple FBDG documents in some countries could affect comprehensiveness, as discrepancies in values across documents existed, which required certain assumptions to be made.

In conclusion, our findings highlighted regional commonalities and disparities in intake recommendations within FBDGs. Disparities were revealed particularly in the provision of quantitative intake guidance. Variability was also observed in how food groups were categorized, influenced by cultural and regional dietary practices. The inconsistent terminology and varied derivation methods further complicate the interpretation of these guidelines and the identification of the key drivers of their variation. Future work should assess whether PS significantly differ across regions. Harmonized efforts are needed for the creation of updated clearer, practical PS guidance for consumers, to enhance adherence to FBDGs.

## Data Availability

The original contributions presented in the study are included in the article/Supplementary material, further inquiries can be directed to the corresponding authors.
