# Corrigendum: A global analysis of portion size recommendations in food-based dietary guidelines

**DOI:** 10.3389/fnut.2025.1548350

**Published:** 2025-02-05

**Authors:** Fanny Salesse, Alison L. Eldridge, Tsz Ning Mak, Eileen R. Gibney

**Affiliations:** ^1^UCD Institute of Food and Health, University College Dublin, Dublin, Ireland; ^2^Insight Centre for Data Analytics, University College Dublin, Dublin, Ireland; ^3^Nestlé Institute of Health Sciences, Nestlé Research, Lausanne, Switzerland; ^4^Nestlé Institute of Health Sciences Singapore Hub, Nestlé Research, Singapore, Singapore

**Keywords:** food-based dietary guidelines, portion size, dietary recommendations, dietary habits, food groups, healthy diet

In the published article, there was an error in Table 6 as published. The recommended portion size for Meat in the Italian food-based dietary guidelines was incorrectly reported as 200g instead of the actual value of 100g stated in the document. Consequently, the maximum recommended portion size for Meat in Europe has been revised to 135g.

The corrected Table 6 and its caption appear below.

**Table 6:** Portion size recommendations in grams for Meat, fish & eggs and PULSES in FBDGs, by FAO region.

		**North America**	**Africa**	**Europe**	**Asia and the Pacific**	**LAC^1^**	**Middle East**	**All**
Meat^3^ (g)	*n* FBDGs	1	6	20	9	14	3	53
	Median	28.4	77.7	92.5	72.5	66.7	30.0	75.0
	SEM^2^	-	2.1	6.5	7.0	5.5	15.0	3.6
	Min	-	69.8	27.5	30.0	30.0	30.0	27.5
	Max	-	85.0	135.0	82.0	98.0	75.0	135.0
Fish & shellfish^3^ (g)	*n* FBDGs	1	6	23	9	9	3	51
	Median	28.4	98.1	120.0	70.6	38.1	75.0	90.0
	SEM	–	18.7	9.9	11.4	8.1	18.0	6.8
	Min	–	58.3	27.5	30.0	30.0	30.0	27.5
	Max	–	190.0	200.0	132.4	90.0	90.0	200.0
Eggs^4^ (g)	*n* FBDGs	1	6	18	8	12	2	47
	Median	50.0	65.0	52.5	50.0	50.0	50.0	50.0
	SEM	–	9.4	6.5	10.7	0.3	0.0	3.4
	Min	–	50.0	50.0	50.0	47.0	50.0	47.0
	Max	–	100.0	125.0	120.0	50.0	50.0	125.0
Pulses^5^ (g)	*n* FBDGs	1	7	20	9	13	3	53
	Median	45.8	95.0	127.5	100.0	80.3	90.7	92.5
	SEM	–	7.9	13.9	24.5	8.6	15.1	7.6
	Min	–	75.0	46.0	30.0	46.0	46.0	30.0
	Max	–	132.0	250.0	240.0	125.0	91.9	250.0

^1^Latin America and the Caribbean.

^2^Standard error of the mean.

^3^Values can be either cooked or raw, depending on the FBDGs. Most of the times no precision was provided as to raw or cooked.

^4^Eggs: 1 medium egg was considered to weigh 50 g.

^5^Pulses: values were considered cooked, except for when “dry” seemed to refer to raw rather than to opposite of fresh pulses (Switzerland), or when the value provided was deemed irrational to be cooked as very low (Estonia). In these cases they were converted to cooked using factor 2.5. Excludes soy products such as tofu, tempeh, etc.

A correction has been made to *Section 3.4.4. Meat, fish & eggs and Pulses*. A sentence previously stated:

“The range of recommended PS values was particularly wide in Europe for Meat (from 27.5 g in Portugal to 200 g in Italy) and Fish & shellfish (from 27.5 in Portugal to 200 g in Romania). The highest PS recommendation for Meat was that of the Italian FBDGs, at 200 g. In regard to Fish & shellfish, maximum amounts were given in the Republic of Moldova and Romania (200 g).”

The corrected sentence appears below:

“The range of recommended PS values was particularly wide in Europe for Meat (from 27.5 g in Portugal to 135 g in Greece) and Fish & shellfish (from 27.5 in Portugal to 200 g in Romania). The highest PS recommendation for Meat was that of the Greek FBDGs, at 135 g. In regard to Fish & shellfish, maximum amounts were given in the Republic of Moldova and Romania (200 g).”

A correction has been made to **Discussion**, paragraph five. The sentence previously stated:

“In Europe for example, the Italian and the Dutch FBDGs extensively mentioned sustainability, however provided recommended PS for Meat of 200 and 100 g, respectively.”

The corrected sentence appears below:

“In Europe for example, the Italian and the Dutch FBDGs extensively mentioned sustainability, however both provided recommended PS for Meat of 100 g, which is greater than the global median.”

In Supplementary Table 3. Portion size recommendations in grams for major food groups in FBDGs, by country, the value for Meat (g) in Italy was reported as 200 instead of 100. The Supplementary Table 3 has been updated.

The authors apologize for this error and state that this does not change the scientific conclusions of the article in any way. The original article has been updated.

